# Factors determining the quality of life of homeless people staying in support centers for people in the crisis of homelessness. Pilot study

**DOI:** 10.1186/s12889-024-17839-w

**Published:** 2024-02-01

**Authors:** Jakub Konrady, Dorota Talarska

**Affiliations:** Department of Preventive Medicine, University of Medical Sciences, Poznan, Poland

**Keywords:** Quality of life, Homelessness, Homelessness Crisis

## Abstract

**Background:**

The aim of the study was to discuss the issues of the homelessness crisis and to present the assessment of the quality of life of people experiencing a homelessness crisis, taking into account various aspects of life and everyday functioning.

**Methods:**

This was a pilot cross- sectional study carried out using an anonymous survey. The author’s questionnaire, the WHOQOL-Bref scale and the Beck depression scale were used. From among the support centers for people in the homelessness crisis operating in the city of Poznań, the 2 largest centers were selected. The obtained results were based on the statistical analysis of the collected data.

**Results:**

The study group consisted only of people in the crisis of homelessness staying at the support centers at the time of the study. The analysis included data from 114 people, including 28 (24.6%) women. The youngest participant was 21 and the oldest 76 years old. The average period of homelessness was 86 months. 55.3% of respondents showed symptoms of depression. The main cause of homelessness was their family situation (59.6%), financial problems (36.0%) and the need to leave the apartment (13.2%). Abuse of alcohol before the homelessness crisis was reported by 96 (84.2%) respondents. The WHOQOL– Bref questionnaire was used to assess the quality of life. The psychological domain was rated the highest (62.09 ± 16.94 points, the lowest somatic domain (53.25 ± 18.71 points). The quality of life of homeless people was positively related to their economic situation, depression and health status. It was shown that sex, age and education had no influence (*p* > 0.05) on the assessment of the quality of life of people experiencing the crisis of homelessness.

**Conclusions:**

The economic situation is the main factor affecting the quality of life within the psychological and social domain. Health status is the main factor affecting the quality of life within the somatic and environmental domain. The biggest dream of the respondents was to have a flat and improve their financial situation.

## Background

The definition of homelessness explains that it is a situation of a person who, at a given time and with the help of external efforts, cannot provide himself with a shelter that he would consider his own, and at the same time this room should meet the minimum conditions to be accepted as living space. People living in institutional centers for the homeless, despite having a place of residence, are also homeless [1]. According to the definition, based on the European Typology of Homelessness and Housing Exclusion (ETOS) - homeless people are both people living in assistance centers and people using non-residential facilities as a permanent shelter, despite having a registered address [2]. Homelessness is a serious social and health problem perceived as a traumatic event. It contributes to the deterioration of health, social isolation, and increases the risk of death [3, 4]. People in the crisis of homelessness are exposed to physical and psychological violence, sexual abuse and persecution. The situation in which they find themselves deprives them of a sense of security and hope for a better future [5]. This situation is conducive to establishing contacts with the criminal environment, dangerous sexual contacts and abuse of addictive substances, especially alcohol and drugs [3].

The number of homeless people in the world is increasing yearly. The reasons are growing economic problems and various social phenomena causing the deterioration of the living situation of the society namely addictions, unemployment, lack of financially accessible housing, family conflicts and mental illnesses [5–8].. The last study conducted in Poland by the Ministry of Family and Social Policy was carried out in February 2019. At that time, there were 30,330 homeless people, the vast majority of whom, over 83%, were men. More than 80% of the surveyed people stayed in institutional facilities. The results showed a decrease in the number of homeless people in the group of women and men compared to 2017 [9]. Regardless of the source, the collected data is described as more or less reliable [7, 8]. The difficulty in accurately recording the number of homeless people is related not only to the inability to reach all homeless people at a given moment, but also to the duplication of data. In addition, the sense of social inferiority and the phenomenon of marginalization do not favor the willingness to participate in social research [1]. For this reason, the given data are inconsistent and make it difficult to compare the scale of the problem in Poland and in other countries. Nevertheless, the phenomenon of homelessness concerns about 0.1% of the population in Poland, while in the world this number varies from 0.1 to 12,5% depending on the country [7, 8].

Regardless of the statistics, homelessness is a serious, often underestimated problem that can affect anyone [6]. When analyzing the causes of homelessness, it can be concluded that almost everyone is at risk of homelessness at some point in their lives.

Most countries, including Poland, do not have national strategies of assistance in the homelessness crisis, and social assistance is implemented differently in each country [8, 9]. The reason for less interest is that homelessness is not perceived as a social problem [6]. The main source of assistance in Poland for people in a difficult life situation (homelessness, unemployment) is the social assistance system. As part of individual programs, homeless people can take advantage of: living in a center or shelter, receiving treatment for addictions, obtaining employment, legal and psychological consultations. The activities of the centers are supported and complemented by associations and non-governmental organizations, e.g. Monar, Caritas, the Saint Albert Aid Society. Actions taken to help people in crisis of homelessness usually do not bring satisfactory results, because the assistance often does not reach those most in need or is insufficient, and after its completion, people return to their previous habits [6, 9]. The reason being the type of projects implemented, as most often they are focused on current social support and not on improving future quality of life.

The quality of life of people experiencing homelessness is influenced by many factors, the most frequently mentioned are: gender; presence of depression; health status, social contacts, length of homelessness and economic situation [10, 11]. Social acceptance is also important. Homeless people want to be treated like the majority of society. However, they face social isolation. Negative attitudes are often perpetuated through films or advertisements showing people in the crisis of homelessness in a light that demeans their dignity, e.g. as garbage collectors or beggars. In reality, homeless people rarely engage in such activities. And persistent stereotypes in society discourage homeless people from admitting to the crisis and seeking help [10, 11].

Previously published works thematically related to homelessness draw attention to the health, legal and social situation of people in the homelessness crisis and the assessment of the quality of life, taking into account physical and mental health, addictions, the ability to meet their needs and the subjective assessment of the quality of life. There is a lack of research assessing the quality of life of people staying in support centers, taking into account demographic variables, economic and family situations and depression. These missing elements were addressed in the pilot study and will be expanded in the main study.

The main purpose of the study was to find out the assessment of the quality of life of people in a homeless crisis, staying in institutions that are part of institutional social welfare, and to check what factors have the greatest impact on their quality of life.

## Methods

### Research Material

Of the 4 support centers for people in crisis of homelessness operating in the city of Poznań, the 2 largest ones (for 106 and 92 people respectively) and located nearby one another were selected. A total of approximately 165 people stayed in both centers during the given period. Other centers can accommodate 30 to 50 people. The research was conducted in the period of May-June 2021, after most of the restrictions due to SARS-CoV-2 coronavirus infection were lifted.

The author visited these centers and each time, after obtaining the consent of the administration of the center, he conducted a survey using the survey method. Each resident of the center who wanted to take in the study, after meeting the inclusion criteria could participate.

Inclusion criteria:

1. Active homelessness crisis - staying and living in a help center for the homeless;
2. Cognitive ability to complete the questionnaire independently or with the help of a researcher;.

Exclusion criteria:

1. The presence of diseases that make it impossible to reliably complete the questionnaire.
2. Significantly developed somatic disease, preventing independent functioning.

Consent for the study was given by the respondent each time before completing the questionnaire after reading the information on the first page. The respondents were each time informed by the researcher about the voluntary completion of the questionnaire, as well as the possibility of withdrawing from the study until the questionnaire was collected. The final completion and return of the questionnaire was tantamount to consent to participate in the study. 125 surveys were collected. 11 questionnaires were excluded from the study: 9 - data gaps preventing any interpretation; 1 - selection of the answer inconsistent with the instructions; 1 - illegible deletion of the answer. 114 questionnaires were included in the study - fully and correctly completed, enabling further analysis.

### Research Organization

This was a pilot cross- sectional study carried out using an anonymous survey. This study aimed to better understand the specificity of final study population. The researcher personally presented the order of the study, then distributed the questionnaires, which were completed by the subjects themselves. At the time of collecting the questionnaires, he checked the completeness of the answers provided and asked for any gaps to be filled in. In case of not understanding the command or difficulties in writing, he was helpful. The data contained in the collected questionnaires were processed by the researcher in accordance with the procedures resulting from the transcription of raw data into data appropriate for the scales used. Then, the collected and ready data were transferred to the database, which was used to carry out statistical analyses.

### Research tools

1. Self-constructed survey, enabling the collection of demographic and social data, including aspects related to the homelessness crisis, e.g. causes of homelessness, duration, etc.
2. WHO QOL - Bref is a shortened questionnaire for assessing the quality of life developed by a team of specialists as part of their work at WHO. The Polish version of the questionnaire is available on the WHO website. The reliability of the questionnaire in the study of the homeless population was checked by, among others, Garcia et al. [12]. The Polish version of the questionnaire was adapted by L. Wołowicka and K. Jaracz from the Department of Nursing, Poznan University of Medical Sciences, Poland. The questionnaire consists of 26 questions, which the respondent answers in relation to their own life situation on a scale of 1–5. The results are obtained by summing up the resulting points from individual questions in 4 domains of quality of life: somatic, psychological, social and environmental. The results can then be transformed into a 20-point or 100-point scale, depending on the scale adopted in a given study.
3. Beck Depression Index (BDI)– used to assess the severity of depression symptoms. It consists of 21 items. Each of the items contains statements that are assigned a score of 0–3. The total score is the sum of the points from all answers. In each statement, the tested person can choose only 1 most suitable answer. A score of 0–11 points means no depression, 12–26 points mild depression, 27–49 points moderately severe depression and 50–63 points very severe depression [13].

The study was approved by the Bioethics Committee at the Medical University of Karol Marcinkowski in Poznań, Poland. The Commission also confirmed that the study does not bear the characteristics of a medical experiment (2019).

### Statistical analysis

The IBM SPSS Statistics 25 package was used for the statistical analyses. All descriptive statistics were analyzed in this package. Depending on the type of variables, appropriate descriptive parameters were used. Measurable variables were described with such parameters as: arithmetic mean, standard deviation (SD), minimum and maximum value. Qualitative variables were described by number (n) and frequency (%).

The following tests were used to test measurable variables:

1. The Kolmogorov-Smirnov test was used to assess the normality of the distribution of the studied interval variables;
2. The significance of differences between more than 2 groups was checked using the Kruskal-Wallis test, and in the event of statistically significant differences, a post-hoc analysis was performed using Dunn’s test. For the two unrelated groups, the Mann-Whitney U test was used;
3. Correlation analyses were carried out using the following coefficients: Pearson’s linear correlation between quantitative variables (age variable) and Spearman’s rs ranks between variables measurable on an ordinal or interval scale without compliance with the normal distribution (for the variables: education, length of the homelessness crisis, economic situation, depression level, health level);

Multiple regression analysis was performed on variables with statistically significant results for each domain of quality of life.

The level of statistical significance of *p* < 0.05 was assumed for all analyses.

## Results

### Characteristics of the study group

The study group consisted only of people in the crisis of homelessness staying at the support centers at the time of the study. Surveys were included in the analysis of 114 people, including 28 (24.6%) women, the youngest participant was 21 and the oldest 76 years old (Table 1). The average age was 55.9 ± 12.1 years.

In the study group, most people had vocational (45.6%) and secondary (27.2%) education. 1/5 of the study group (21.1%) had only primary education. The average period of homelessness was 86 months. Most of the respondents maintained social contacts with their families (43.0%). On the other hand, 1/4 (24.6%) of the group was only surrounded by people in the place of residence. More than half (53.5%) assessed their economic situation as neither good nor bad. 55.3% of respondents showed symptoms of depression seen in the Beck Depression Index, where they scored from 12 to 40 points. 36.0% of the group were satisfied with their health and 40.3% were dissatisfied. The following diseases were most frequently reported by the respondents: hypertension (*n* = 90, 78.9%), degenerative changes of the spine (*n* = 56, 49.1%), insomnia (*n* = 14, 12.3%), mental illness (*n* = 5, 4.4%). Almost all people included in the study smoked cigarettes (*n* = 103, 90.4%). One person admitted to gambling.

**Table 1:** Characteristics of the study group

Variable		*N* = 114	100%
Gender	Female	28	24.6
	Male	86	75.4
Age	Up to 40 years old	13	11.4
	41–59 years old	47	41.2
	Over 60 years old	54	47.4
Education	Primary	25	21.9
	Vocational	52	45.6
	High School	31	27.2
	Higher	6	5.3
Stay length in the homelessness crisis	Up to 86 months	78	68.4
	Over 86 months	36	31.6
Social contacts	Family	49	43.0
	Friends	21	18.4
	Family and friends	16	14.0
	None	28	24.6
Economic situation	Very poor	15	13.2
	Poor	17	14.9
	Neither poor nor good	61	53.5
	Good	20	17.5
	Very good	1	0.9
Beck Depression Index	0–11 pts.– no depression	51	44.7
	12–26 pts.– mild depression	49	43.0
	27–49 pts.– moderately severe depression	14	12.3
Health Satisfaction	Dissatisfied	46	40.3
	Neither dissatisfied nor satisfied	27	23.7
	Satisfied	41	36.0

The main cause of homelessness was their family situation (*n* = 68, 59.6%), financial problems (*n* = 41, 36.0%) and the need to leave the apartment (*n* = 15, 13.2%). Abuse of alcohol before the homelessness crisis was reported by 96 (84.2%) respondents. After the homelessness crisis, 4 (3.5%) men admitted to drinking alcohol. The current source of income for the majority (*n* = 112, 98.2%) was the benefit paid by the Municipal Family Support Center (MOPR). 9 (7.9%) people received a pension.

A chance to get out of homelessness was seen by 108 (94.7%) respondents. Most of them expected help from their family (*n* = 46, 40.4%) or state institutions (*n* = 85, 74.6%).

They expected higher benefits from the state (77.2%), housing (41.2%) and employment (23.7%). On the other hand, psychological support (65.8%), love (40.4%), help in finding a job and a flat (10.5%) came from the family. Among the dreams mentioned by the study participants, they mainly mentioned having: a flat (93.9%), a job (64.9%), a normal family (13.2%), health (10.5%) and good upbringing of children (7.0%).

### Quality of life

The WHOQOL– Bref questionnaire was used to assess the quality of life. The psychological domain was rated the highest (62.09 ± 16.94 points), followed by the social domain (56.17 ± 21.76 points) and the environmental domain (56.91 ± 16.17 points), the lowest somatic domain (53.25 ± 18.71 points).

In order to determine the factors influencing the assessment of the quality of life, basic demographic and social data were taken into account in the analysis. Although the study group was diverse in terms of age, gender and duration of homelessness, it was shown that gender, age, education, length of stay in the homelessness crisis and maintaining social contacts do not influence the assessment of the quality of life of people experiencing the homelessness crisis. In all domains of quality of life, the results were not statistically significant (*p* > 0.05).

The variable that showed a significant relationship with all areas of the quality of life was the economic situation: somatic (rs 0.33 *p* < 0.001), psychological (rs 0.39 *p* < 0.001), social (rs 0.41 *p* < 0.001), environmental (rs 0.40 *p* < 0.001). The assessment of the quality of life increased with the level of economic situation. The strength of the observed associations was moderately high.

In the next step, it was checked whether the level of respondents’ depression was related to the level of quality of life. A series of Spearman’s rs rank correlation analyses were performed, and all relationships turned out to be statistically significant (*p* < 0.05). The level of depression was negatively associated with the level of quality of life in all four domains. The assessment of the quality of life decreased as the level of depression of the surveyed people increased. The strength of the correlation between the level of depression and the quality of life in the somatic domain was high (rs -0.56), the remaining three relationships were moderately strong (psychological rs -0.49; social rs -0.34; environmental rs -0.38).

Health status also had a significant impact on the assessment of the quality of life in the surveyed group of people in the homeless crisis. In each of the four domains (somatic rs 0.70 *p* < 0.001; psychological rs 0.54 *p* < 0.001; social rs 0.47 *p* < 0.001; environmental 0.55 *p* < 0.001) the level of health satisfaction was positively related to the assessment of quality of life. The rating increased along with the feeling of better health. In terms of the social domain, the strength of this impact was moderately high, while for the other three domains it was high.

In order to determine the factor most significantly influencing the assessment of the quality of life of the homeless, multiple regression was performed for individual domains of the quality of life (Tables 2 and 3).

**Table 2:** Multiple regression results for the quality of life in the somatic and psychological domains

Variables	Somatic Domain			Psychological Domain		
	b* ± SD	t(105)	p	b* ± SD	t(105)	p
Free choice		3.89	**< 0,001**		4.11	**< 0.001**
Gender	0.091 ± 0.066	1.39	0.168	-0.025 ± 0.075	-0.33	0.745
Age	0.030 ± 0.066	0.46	0.648	0.105 ± 0.076	1.38	0.170
Education	-0.072 ± 0.065	-1.10	0.275	0.032 ± 0.075	0.42	0.673
Homelessness	-0.008 ± 0.066	-0.13	0.899	-0.092 ± 0.075	-1.22	0.226
Social contacts	-0.082 ± 0.065	-1.26	0.210	-0.068 ± 0.074	-0.92	0.361
Economic situation	0.058 ± 0.069	0.83	0.407	0.230 ± 0.079	2.90	**0.005**
Depression	-0.393 ± 0.073	-5.35	**< 0.001**	-0.410 ± 0.084	-4.88	**< 0.001**
Health status	0.496 ± 0.078	6.35	**< 0.001**	0.271 ± 0.089	3.03	**0.003**

b*Standardized error ± standard deviation (SD) of the standardized error, Statistically significant result, *p* < 0.05, is marked

The following variables turned out to be significant for the quality of life in the somatic domain: depression (*p* < 0.001) and health status (*p* < 0.001). The regression model explains 59.9% of the variation in quality of life in the somatic domain. Health status had a greater impact on the quality of life in the somatic domain than the level of depression.

The following variables turned out to be significant for the quality of life in the psychological domain: economic situation (*p* = 0.005), depression (*p* < 0.001) and health status (*p* = 0.003).

The regression model explained 47.4% of the variability in quality of life in the psychological domain. The increase in depression, worse economic situation and worse health condition in the respondents caused a decrease in the quality of life in the psychological domain. The greatest impact on the quality of life in the psychological domain had, in turn, the following: economic situation, health status and level of depression.

**Table 3:** Multiple regression results for quality of life in the social and environmental domains

Variables	Social domain			Environmental domain		
	b* ± SD	t(105)	p	b*± SD	t(105)	p
Free choice		3.04	**0.003**		2.31	**0.023**
Gender	0.027 ± 0.088	0.31	0.758	-0.018 ± 0.076	-0.23	0.815
Age	-0.079 ± 0.088	-0.90	0.372	0.232 ± 0.077	3.03	**0.003**
Education	-0.022 ± 0.088	-0.25	0.805	-0.071 ± 0.076	-0.94	0.352
Homelessness	-0.018 ± 0.088	-0.20	0.840	-0.146 ± 0.076	-1.92	0.058
Social contacts	-0.052 ± 0.087	-0.60	0.547	-0.047 ± 0.075	-0.62	0.535
Economic situation	0.218 ± 0.092	2.36	**0.020**	0.244 ± 0.080	3.04	**0.003**
Depression	-0.249 ± 0.098	-2.54	**0.012**	-0.192 ± 0.085	-2.26	**0.026**
Health status	0.248 ± 0.104	2.38	**0.019**	0.411 ± 0.091	4.54	**< 0.001**

b* Standardized error ± standard deviation (SD) of the standardized error, Statistically significant result, *p* < 0.05, is marked

The following variables turned out to be significant for the quality of life in the social domain: economic situation (*p* = 0.020), depression (*p* = 0.013), and health status (*p* = 0.019). The regression model explained 28.4% of the variation in quality of life in the social domain. The greatest impact on the quality of life in the social domain had, in turn, the following: economic situation, health status and level of depression. The increase in depression, worse economic situation and worse health condition in the respondents caused a deterioration in the assessment of the quality of life.

The following variables turned out to be significant for the quality of life in the environmental domain: age (*p* = 0.003), economic situation (*p* = 0.003), depression (*p* = 0.026), and health status (*p* < 0.0001). The regression model explained 46.2% of the variation in quality of life in the environmental domain. Younger age, increased depression, worse economic situation and poorer health in the respondents caused a decrease in the quality of life in the environmental domain.

The greatest impact on the quality of life in the environmental domain had, in turn, health status, economic situation, age and level of depression.

## Discussion

The group covered by the study represented quite characteristic features: they were mostly middle-aged men with vocational education. However, quite a large group was also made up of people with primary and secondary education. Additionally, they lived in support centers. Similar features were also noted by other authors [3, 5, 11, 14–16]. The specificity of the group translated into the obtained research results.

The quality of life was assessed as quite good, especially in the psychological area, but lower in other domains. Due to the occurrence of various health disorders, the somatic domain was rated the lowest. The quality of life was generally assessed by people in the homelessness crisis at a fairly good level also in other studies [3, 4]. Usually the social domain was ranked lowest [17, 18]. Perhaps the result in the Polish group was due to the fact that they were residents of support centers for the homeless.

The most frequently indicated factors in the literature review that affect the quality of life of homeless people were: poorer health, especially mental disorders such as depression or schizophrenia, having few or no social contacts, lack of prospects for the future, low social status and engaging in criminal activity, use of psychoactive substances, lack of housing and income [3, 14, 15]. Health status was one of the more frequently mentioned factors significantly influencing the quality of life of adults [,4, 10,11,14,16,19]. In the study group, 40.3% of people were dissatisfied with their health due to the presence of multiple diseases and depression. Homeless people often suffer from diseases of the musculoskeletal system, sleep disorders, respiratory and circulatory system diseases, mental illnesses, and infectious diseases such as tuberculosis, hepatitis C, AIDS, and dental neglect [4, 14]. Similar disorders were noted in the group included in our study. The dominant mental health problem was depression. Other authors also point out that depression and other mental disorders negatively affected the assessment of quality of life [5, 11].

Demographic factors and the level of health care were also factors that significantly influenced the quality of life of homeless people. Although previously published results contained differing data, lower levels of education and poorer access to health care contributed to low assessment of quality of life [10, 11, 18, 19]. However, older people, women, people without a criminal record, with a shorter period of homelessness, and staying in Housing First (HF) support centers usually had a higher quality of life [11]. In our own research, gender, age, education and length of stay did not have a significant impact on the quality of life of people in the crisis of homelessness. Respondents in our own study were a heterogeneous group in terms of the length of their stay in the homeless crisis. The shortest recorded time of being homeless was 1 month, while the longest was 636 months. The diversity of the group may have influenced the final results of the analysis.

Another factor analysed was social contacts. In our group, there was no significant connection between social relations and the assessment of quality of life. The respondents most often kept in touch with family or friends. Perhaps staying in the center and constant contact with others satisfied their need for interpersonal relationships. Other authors also emphasize that maintaining social contacts facilitates receiving appropriate support and has a positive impact on the assessment of the quality of life [3, 19, 20].

In order to identify factors that significantly influence the quality of life of people in the crisis of homelessness, the next stage was to conduct a regression analysis. The multiple regression models developed by us showed a significant relationship between the assessment of the quality of life and the severity of depressive symptoms and the assessment of health status in all domains and additionally the economic situation (except the somatic domain) and only in the environmental domain with age. Reports by other authors have also shown a link between the economic situation and the assessment of the quality of life [5, 19]. The lack of a stable income and one’s own place of residence contributed to the disturbance of the need for security. This negative feeling was strengthened by the loss of professional activity and income, stigmatization of the environment and not seeing the possibility of changing the situation [5]. It could also be a contributing factor for the development of depression [11].

The general life situation of people experiencing the crisis of homelessness is not conducive to maintaining a high quality of life, and yet the conducted research showed that homeless people assessed their quality of life at an average level [10, 11, 18].

Homeless people cope with ad hoc help offered to them. They often live from day to day, do odd jobs, use social assistance or engage in criminal activity (contrary to appearances, this is a rarity). Their behavior most often depends on what stage of entering homelessness they are at. In the initial period of homelessness, they still hope to get out of the crisis quickly, so they try to change something and remain independent [21].

As such, the way of coping during the homelessness crisis largely depends on the ability to cope with difficult situations, i.e. the adopted adaptation mechanisms [3]. Good strategies make it easier to adapt to difficult situations, protect physical and mental health and mobilize to take action to improve the situation. People with higher adaptive skills usually show a higher quality of life [3, 16]. Positive adaptation is conducive to good social relations, received support and a safe place to stay, e.g. in support centres, lack of mental disorders and addictions [3]. In our own research, the majority of people, regardless of the length of being in the homeless crisis, dreamed of an independent apartment. There is a shortage of affordable housing in Poland and subsequently most families cannot afford or pay off the cost of a mortgage [8, 9]. Such a situation favors homelessness and hinders getting out of homelessness [14]. Yet regardless of how homeless people assessed their quality of life, most of them wanted to become independent [5].

In order to improve the quality of life of homeless people, appropriately planned social policy programs should be implemented, focused on the current analysis of the needs of homeless people [6, 11, 18, 19]. Programs should be flexible enough to provide the optimal type of support. Referring to the obtained results, priority should be given to programs enabling the acquisition of social housing and re-activation in the labor market, which could be a source of income and make it possible to pay for the flat of their dreams. Meetings with a psychologist or even a personal trainer are also important, with whom it would be possible to discuss the stages of overcoming the homelessness crisis. Also, the health care sector should be better prepared to provide services to people in the homeless crisis. Not only by providing professional care but also, and perhaps above all, by the appropriate approach of the staff, in order to reduce the feeling of exclusion and social marginalization among the homeless. The priority is the need to develop the support offered to the homeless by basic health care. It should become the first link in providing medical assistance, as well as in the field of prevention. Moreover that a significant part of the problems experienced is closely related to deteriorating health. Dental care should also be provided to the homeless.

It is also necessary to properly prepare society so that it better understands the specifics of the homelessness crisis and is able to counteract it more effectively. Especially in relation to people with addictions and suddenly losing their jobs. This will help combat exclusion and social marginalization, which are among the most frequently reported problems by homeless people [10, 11, 18, 19].

### Limitations of the study and future perspectives

The main limitation of the conducted research was the small group of people in crisis of homelessness, which contributed to the large diversity of the group. Additionally, this was a group staying in support centers where the condition of stay was not consuming alcohol. The specificity of the group could have influenced the perception of the situation of the homeless. In the planned actual study, the study group should be expanded to include homeless people from other facilities and those who do not use institutional shelter. It is also worth enriching the quantitative research with a qualitative one. Despite the above-mentioned limitations, the results obtained in the pilot study show the most common problems in the group of people in the midst of a crisis of homelessness.

## Conclusion

In the pilot study, people in a crisis of homelessness rated the psychological domain the highest and the somatic domain the lowest. This indicates the need to increase programs aimed at improving the health of people in the homeless crisis. In the study group, the quality of life was positively related to their economic situation, depression and health status. In the psychological and social domain, the main factor influencing the quality of life was their economic situation, while in the somatic and environmental domain, their current health status was the main factor. The importance of the economic situation is reflected in the dreams of homeless people. The biggest dream of the people surveyed was to have an apartment and improve their financial situation. Both factors were intended to ensure the independence of people affected by the homelessness crisis and thus improve quality of life.

## Data Availability

The datasets used and/or analysed during the current study are available from the corresponding author on reasonable request.
